# Characterization of the complete chloroplast genome of *Lycium ruthenicum* (Solanaceae)

**DOI:** 10.1080/23802359.2018.1450681

**Published:** 2018-03-22

**Authors:** Gulbar Yisilam, Reyim Mamut, Jin Li, Pan Li, Chengxin Fu

**Affiliations:** a Xinjiang Key Laboratory of Special Species Conservation and Regulatory Biology, Key Laboratory of Plant Stress Biology in Arid Land, College of Life Science, Xinjiang Normal University, Urumqi, China;; b Key Laboratory of Conservation Biology for Endangered Wildlife of the Ministry of Education, and Laboratory of Systematic & Evolutionary Botany and Biodiversity, College of Life Sciences, Zhejiang University, Hangzhou, China

**Keywords:** Anthocyanin, chloroplast genome, phylogeny, medicinal plant

## Abstract

*Lycium ruthenicum* is a precious desert plant with high medicinal and ecological values. It contains rich anthocyanins, which are safe natural pigments and could be used for natural food colorants. In this study, the complete chloroplast (cp) genome of *L. ruthenicum* was assembled on the basis of next generation sequencing. The whole genome is 154,996 bp in length, including two single copy regions (LSC: 85,993 bp and SSC: 18,213 bp) and two inverted repeat regions (IR: 25,395 bp). The overall G + C content of the genome is 37.9%. The cp genome consists of 111 unique genes, including 30 tRNA genes, 4 rRNA genes and 77 protein-coding genes. Phylogenetic analysis indicates that *L. ruthenicum* is closely clustered with *Atropa belladonna*, *Przewalskia tangutica*, and *Scopolia parviflora*.

The genus *Lycium* contains several most well-known medicinal and food plants in Solanaceae, such as *L. barbarum*, *L. chinense*, and *Lycium ruthenicum*. There are about 80 *Lycium* species around the world (Hitchcock 1932; Miller 2002; Levin and Miller 2005), with only seven species been found in China. Among them, *L. ruthenicum* is very important for the ecosystem because it is resistant to drought and salt stress and could reduce soil salinity (Zheng et al. 2011). It is also used to treat heart disease, ureteral stones, tinea, furuncle and gingival bleeding (Liu et al. 2014). Besides, the fruits of *L. ruthenicum* contain rich anthocyanins, which are safe natural pigments and could be used as food colorants. Recent studies have reported that the wild resources of *L. ruthenicum* rapidly declined due to human disturbance. Thus, it has been listed among the Key Protected Wild Plants (Category II) in Qinghai Province (http://www.qhys.gov.cn/html/42/202883.html). To scientifically and effectively conserve this plant, collecting more genetic information is important. Complete chloroplast (cp) genome could be applied to studies on population genetics and phylogenetics. It is useful to discuss the occurrence, evolution, and conservation of this precious endangered species.

Fresh and healthy leaves samples of wild *L. ruthenicum* were collected from the Yuepuhu County in Xinjiang Autonomous Region of China (39°12′39.6″N, 76°46′20.5″E,). Voucher herbarium specimens (GY17001) were deposited at the Herbarium of Xinjiang Normal University (XJNU). The total DNA was extracted using DNA Plantzol Reagent (Invitrogen, Shanghai, China) according to the manufacturer’s protocol, and then sequenced using Illumina HiSeq^TM^ 2000 (San Diego, CA). The chloroplast sequences were assembled by NOVOPlasty 2.6.3 (Dierckxsens et al. 2017) with complete chloroplast sequence of *Atropa belladonna* (GenBank accession number: NC004561) as the reference. Finally, the whole sequence was annotated with Geneious 11.0.2 (Biomatters Ltd., Auckland, New Zealand). Then the complete circle was accomplished by OGDRAW (http://ogdraw.mpimp-golm.mpg.de/). The complete cpDNA sequence has been submitted to GenBank (MG976805). *Lycium ruthenicum* sequence was also aligned with the other Solanaceae cp genomes. The phylogenetic tree was constructed by MEGA6 (Tamura et al. 2013), with a Convolvulaceae species as the outgroup.

The whole genome of *L. ruthenicum* is 154,996 bp in length, including a large single copy region (LSC: 85,993 bp), a small single copy region (SSC: 18,213 bp) and two inverted repeat regions (IR: 25,395 bp). The overall G + C content of the genome is 37.9%. The cp genome consists of 111 unique genes, including 30 tRNA genes, 4 rRNA genes and 77 protein-coding genes. Seventeen genes are duplicated in the IR regions, including all four rRNA genes, seven tRNA genes and six protein-coding genes. Sixteen genes have one intron, while two genes have two introns. The *ycf1*, *rps19* genes are crossing the SSC/IR and LSC/IR borders. The *rps12* gene is trans-spliced, with the 5′ end located in the LSC and the 3′ end duplicated in the IR regions.

To figure out the phylogenetic position of *L. ruthenicum* within Solanaceae, we constructed neighbour joining (NJ) tree (with 1000 bootstrap replicates) using all Solanaceae species which have publicly available cp genome, with one species of Convolvulaceae (*Ipomoea purpurea*: NC009808) as the outgroup. It is found that *L. ruthenicum* is sister to a clade of *Atropa belladonna*, *Przewalskia tangutica*, and *Scopolia parviflora* (Figure 1). In conclusion, this is the first research article of the complete cp genome of the *L. ruthenicum*, and this information which establish a solid foundation for future conservation genetic and phylogenetic studies.

**Figure 1.**
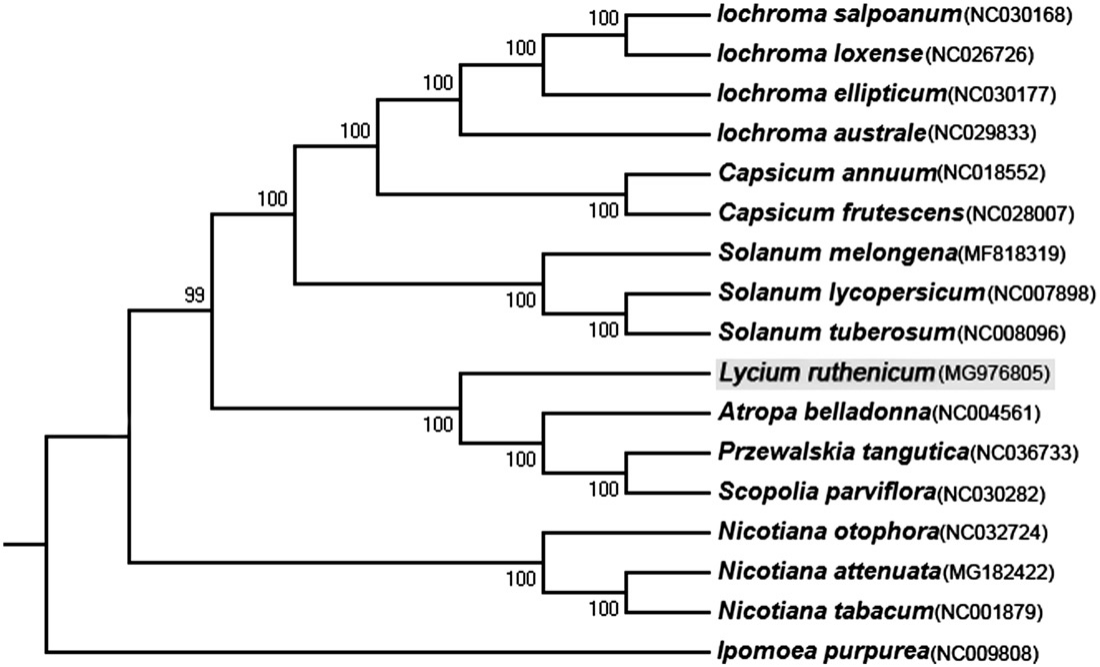
Neighbour-joining phylogeny of Solanaceae based on 17 complete cp genomes. The accession numbers are listed as below: *Iochroma salpoanum* (NC030168), *Iochroma loxense* (NC026726), *Iochroma ellipticum* (NC030177), *Iochroma australe* (NC029833), *Capsicum annuum* (NC018552), *Capsicum frutescens* (NC028007), *Solanum melongena* (MF818319), *Solanum lycopersicum* (NC007898), *Solanum tuberosum* (NC008096), *Lycium ruthenicum* (MG976805), *Atropa belladonna* (NC004561), *Przewalskia tangutica* (NC036733), *Scopolia parviflora* (NC030282), *Nicotiana otophora* (NC032724), *Nicotiana attenuata* (MG182422), *Nicotiana tabacum* (NC001879), *Ipomoea purpurea* (NC009808).
